# Decoupling of arsenic and iron release from ferrihydrite suspension under reducing conditions: a biogeochemical model

**DOI:** 10.1186/1467-4866-8-12

**Published:** 2007-11-29

**Authors:** André Burnol, Francis Garrido, Philippe Baranger, Catherine Joulian, Marie-Christine Dictor, Françoise Bodénan, Guillaume Morin, Laurent Charlet

**Affiliations:** 1 BRGM, Bureau de Recherches Géologiques et Minières, Environment and Process Division, 3, avenue Claude Guillemin, BP 6009, 45060 Orléans Cedex 2, France; 2 IMPMC, Institut de Minéralogie et de Physique des Milieux Condensés (IMPMC), UMR 7590 – CNRS – Universités Paris 6&7 – IPGP, 140, rue de Lourmel, 75015 Paris, France; 3 Environmental Geochemistry Group, LGIT-OSUG, Grenoble University, 38041 Grenoble Cedex 9, France

## Abstract

High levels of arsenic in groundwater and drinking water are a major health problem. Although the processes controlling the release of As are still not well known, the reductive dissolution of As-rich Fe oxyhydroxides has so far been a favorite hypothesis. Decoupling between arsenic and iron redox transformations has been experimentally demonstrated, but not quantitatively interpreted. Here, we report on incubation batch experiments run with As(V) sorbed on, or co-precipitated with, 2-line ferrihydrite. The biotic and abiotic processes of As release were investigated by using wet chemistry, X-ray diffraction, X-ray absorption and genomic techniques. The incubation experiments were carried out with a phosphate-rich growth medium and a community of Fe(III)-reducing bacteria under strict anoxic conditions for two months. During the first month, the release of Fe(II) in the aqueous phase amounted to only 3% to 10% of the total initial solid Fe concentration, whilst the total aqueous As remained almost constant after an initial exchange with phosphate ions. During the second month, the aqueous Fe(II) concentration remained constant, or even decreased, whereas the total quantity of As released to the solution accounted for 14% to 45% of the total initial solid As concentration. At the end of the incubation, the aqueous-phase arsenic was present predominately as As(III) whilst X-ray absorption spectroscopy indicated that more than 70% of the solid-phase arsenic was present as As(V). X-ray diffraction revealed vivianite Fe(II)_3_(PO_4_)_2_.8H_2_O in some of the experiments. A biogeochemical model was then developed to simulate these aqueous- and solid-phase results. The two main conclusions drawn from the model are that (1) As(V) is not reduced during the first incubation month with high Eh values, but rather re-adsorbed onto the ferrihydrite surface, and this state remains until arsenic reduction is energetically more favorable than iron reduction, and (2) the release of As during the second month is due to its reduction to the more weakly adsorbed As(III) which cannot compete against carbonate ions for sorption onto ferrihydrite. The model was also successfully applied to recent experimental results on the release of arsenic from Bengal delta sediments.

## Background

High concentrations of arsenic in natural waters represent a major health problem for humans in many places around the world [1], and particularly in South Asia (West Bengal, Bangladesh) where tens, and possibly hundreds, of millions of people consume groundwater containing arsenic levels exceeding the 10 μgL^-1 ^guideline value defined by the World Health Organization [2]. The processes controlling the solubilization of arsenic have been studied intensively over the last decade [3-6], but still remain a subject of debate [7]. For the Bangladesh and West Bengal aquifers, however, the most widely accepted mechanism for the cause of high aqueous arsenic concentrations is the microbial reductive dissolution of iron oxyhydroxides and concomitant release of arsenic.

The abiotic and biotic processes which could lead to the mobilization of iron-associated arsenic may be intimately coupled. An indirect mechanism of biotic As(V) release has been reported in experiments with synthetic scorodite Fe(III)AsO_4_.2H_2_O in the presence of Fe(III)-respiring *Shewanella Alga*, although this organism did not reduce As(V) [8]. Conversely, an arsenic-resistant *Clostridium*, CN8, is capable of reducing As(V) but not Fe(III) in ferrihydrite, or As(V) adsorbed on ferrihydrite [9]. A direct mechanism of arsenic mobilization has been reported from experiments with As-rich ferrihydrite in the presence of *Sulfurospirillum barnesii*, a bacterium that respires both As(V) and Fe(III) [10]. The authors report the ability of this microorganism to reduce As(V) adsorbed on synthetic ferrihydrite. Only a single strain was used in these experiments, and the key question concerning the role of a diverse community of dissimilatory iron-reducing bacteria (DIRB) in arsenic release was left unanswered.

Recent observations on the incubation of Bengal delta sediments have demonstrated that anaerobic metal-reducing bacteria can play a key role in the mobilization of arsenic. Moreover, the incubation experiments revealed that arsenic and iron release can be decoupled: most of the leachable arsenic, but only a small fraction of the leachable iron, can be released over a 2-month period [11] and arsenic release can appear after Fe(III) reduction, rather than simultaneously [12].

In this paper, we investigate the release of arsenic sorbed on or co-precipitated with iron oxyhydroxides, and more specifically the question of the uncorrelated release of dissolved Fe and As observed both in the laboratory [12] and in the field [13-15]. We discuss the results of batch experiments on the bacterial reduction of synthetic As-rich 2-line ferrihydrite (As-2LFh) by an Fe(III)-reducing bacterial community (FR) that was obtained by enrichment from a soil containing high concentrations of heavy metals (See Additional File 1). The experiments, using microbiology in combination with aqueous- and solid-phase speciation analysis, were carried out under controlled anoxic laboratory conditions over a period of two months.

The objectives of the study were to (1) investigate abiotic and biotic processes in the release of arsenic sorbed on or co-precipitated with ferrihydrite, (2) compare the results with recent observations on the decoupling of As and Fe release after incubation of Bengal Delta Plain sediments [12], and (3) evaluate different explanations of the As release using a biogeochemical model.

## Methods

### Incubation experiments with As-2LFh

The protocols for obtaining synthetic arsenate-doped 2-line ferrihydrite by adsorption (initial AD samples) or co precipitation (initial CP samples) were adapted from standard methods [16,17]. Chlorinated salts were preferred over ferric nitrates in order to avoid any use of nitrates as a growth electron acceptor by bacteria. The conventional steps of centrifugation and washing with MilliQ water to remove chlorides were simplified. A first 0.36 M FeCl_3 _solution (10 L at pH < 2) was prepared in a continuously stirred reactor and labeled "Solution A", and a second 0.22 M Na_2_HAsO_4 _solution (600 ml at pH 8.7) was prepared and labeled "Solution B". The initial AD samples were prepared by adding an appropriate amount of 10 M NaOH solution to 5 L of the continuously stirred Solution A so as to obtain a pH of ~6 and precipitate the hydrous ferric oxide (2LFh). The pH was then maintained constant by automatic titration (Mettler DL21) with a 1 M NaOH solution, and equilibrated for 1 hour. The next step was to add 300 mL of Solution B, regulated to pH ~6 by the addition of a 1 M HCl solution, and continuously stir the mixture for 2 hours. The initial CP samples were prepared by adding 300 mL of Solution B to 5 L of continuously stirred Solution A (at pH 2). The co-precipitation of As-2LFh was achieved by neutralizing this mixture to pH 6, using an appropriate amount of 10 M NaOH. The pH was then maintained constant by automatic titration with a 1 M NaOH solution and equilibrated for 2 hours.

In both preparations, the solids were washed twice with 1 L of deionized water and recovered by Büchner filtration. The initial AD and CP solids had a moisture content of about 85–90 wt.% and were stored at 4°C until used. The As/Fe molar ratio in the freeze-dried solids was about 5.5% (Table 1) as measured after hot HCl extraction [18].

**Table 1:** Solids characterization using hot HCl extraction, XRD and XANES for all experiments at 0 day (initial), 21 days and 63 days (n.d.: no data).

	AD1	CP1	AD5	CP5
Synthesis mechanism	Adsorption	Co-precipitation	Adsorption	Co-precipitation
Initial solid As (mM)^1^	0.17	0.17	0.84	0.84
Initial solid Fe (mM)^1^	3.06	3.06	15.3	15.3
Initial molar solid As/Fe (%)^1^	5.48	5.48	5.55	5.55
Initial solid As(III)/As (%)^2^	< 5	< 5	< 5	< 5
After 21 days solid As(III)/As (%)^2^	9	8	n.d.	n.d.
After 63 days solid As(III)/As (%)^2^	16	n.d.	29	n.d.
Initial solid composition^3^	2LFh	2LFh	2LFh	2LFh
After 21 days solid composition^3^	2LFh	2LFh	2LFh	2LFh
After 63 days solid composition^3^	2LFh, Vivianite, Bobierrite	2LFh, Vivianite	2LFh	2LFh

^1 ^From hot HCl extraction.

^2 ^From XANES (accuracy on the molar As(III)/As ratio is ± 3%).

^3 ^From XRD.

The AD and CP experiments consisted in incubating various amounts of As-2LFh in a growth medium both with and without FR. The experiments were conducted under sterile conditions (three 100°C cycles of one hour at 24-hour intervals) in 600 mL plasma flasks under a CO_2 _atmosphere at 20 °C with continuous stirring for about two months. For the biotic experiments, 50 mL of FR inoculum were added to 450 mL of a selective liquid medium called CAsR1; this growth medium had been used previously for growing arsenic-reducing bacteria [19] and the composition is described in Additional File 1. All biotic experiments were performed in duplicate. The same experiments with 500 mL of culture medium CAsR1, but without bacteria, served as control.

Experiments AD1 and CP1 were essentially identical and used 3.1 mM Fe and 0.17 mM As (respectively adsorbed or co-precipitated). Experiments AD5 and CP5 used 15.3 mM Fe and 0.84 mM As (respectively adsorbed or co-precipitated; see Table 1).

### Sampling and analysis

The supernatant in all the experiments was sampled (10 mL) weekly with a syringe under anaerobic conditions. After filtration at 0.1 μm and acidification with HCl, total iron was analyzed by Atomic Absorption Spectrometry (AAS) (Varian SpectrAA 300 Zeeman) with a detection limit of 0.06 mg L^-1^. The Fe(II) concentration was measured colorimetrically with the ferrozine reagent using an Agilent 8453E UV-Visible [20]. An aliquot of the filtered sample was immediately passed through an anionic exchange column that retains As(V) but not As(III) [21] and, after acidification, analyzed by AAS with a detection limit of 5 μg L^-1^.

The solid concentrations of contained Fe and As were measured after hot HCl extraction [18] and are listed in Table 1. The solid phases were recovered by centrifugation (Jouan CR412, 15 minutes at 6000 rpm) and freeze-dried. The dry solids were maintained under anaerobic conditions until mineralogical characterization.

Microscopic observations and qualitative analyses were performed at the University of Orléans using a Philips CM20 Transmission Electron Microscope (TEM) with a CCD Gatan camera at 200 kV. The TEM samples were prepared by dispersing the powdered samples in alcohol by ultrasonic treatment, dropping them onto a porous carbon film supported on a copper grid, and then drying them in air. Crystalline phases were determined by X-ray powder diffraction (XRD), the XRD data being analyzed using a diffractometer with a Bragg Brentano geometry (Siemens D5000) equipped with a monochromator and based on cobalt K_α1 _radiation (λ = 1.78897 Å). Acquisition time for the XRD patterns in the 4–84°2θ interval was set at one second per 0.02°2θ step (= 1 hour) for rotating samples. The crystalline phases were then identified using Diffrac-AT software in conjunction with the Joint Committee on Powder Diffraction Standards (JCPDS) database. Samples were loaded on aluminum plates.

The arsenic oxidation state was measured by X-ray Absorption Near Edge Spectroscopy (XANES), using the procedure described by Morin et al. [22]. The data were recorded at the As K-edge (11859 eV) at room temperature in transmission mode on the D44 bending-magnet beamline at the LURE (Orsay, France). A Si(511) double-crystal monochromator yielded an energy resolution of approximately 0.5 eV, the energy being calibrated by a double-transmission setup using an Au foil as reference. Summing 6 scans for each sample yielded reliable signal/noise ratios. Proportions of As(III) and As(V) were determined by linear least squares fitting of the XANES spectra using the spectra of two relevant model compounds, cpp3 and cpp5, consisting of Fe(III)-As(III) and Fe(III)-As(V) amorphous hydroxides, respectively. Absolute accuracy on the As(III)/As(total) ratio was ± 3% [22]. According to this calibration procedure, components lower than 5% are not significant. The absence of As(III) oxidation due to the X-ray beam was checked by replicating the spectra at the same points. Taking into account what it is known about the photo-oxidation of As(III) onto iron oxides, only a very weak oxidation was expected in these samples because of the high As/Fe molar ratio (about 5.5%). Moreover, in contrast to the new generation of beamlines, the intensity of the LURE (Orsay) beam was quite low.

Total bacteria were counted under an optical light microscope (Nikon, with magnification ×400) using a Thoma cell. The isolation and identification of pure strains from the FR community with phylogenetic characterization are described in Additional File 1.

### Thermodynamic and kinetic modeling

When redox reactions proceed under thermodynamic equilibrium, the solubility of an Fe(III) oxyhydroxide can be obtained by measuring the redox potential Eh of the suspension. Taking, for example, pure Fe(III) oxyhydroxide

Fe(OH)_3_(*s*) + 3H^+^(*aq*) = Fe^3+^(*aq*) + 3H_2_O(*l*)

and combining this with the redox half-reaction

Fe^3+^(*aq*) + e^- ^= Fe^2+^(*aq*)

we obtain the redox potential Eh (in mV):

$$E_{H}(Fe)=59\times \left(\log(K_{sp}K_{red})-3pH-\log a_{Fe^{2+}}\right)$$

where *K*_sp _is the solubility product for Eq. (1), *K*_red _(= 10^13.05 ^at 25 °C) the equilibrium constant for Eq. (2), and a_Fe2+ _the activity of aqueous ferrous iron.

When the crystal grain is infinitely large, the solubility product *K*_sp _can approximate the crystal solubility *K*_so_. In the case of small particles with a large surface, the solubility *K*_so _of the particles is highly dependent on the surface energy [23]:

$$\log K_{so}=\log K_{sp}+\frac{2}{3}\alpha V\frac{1}{\ln(10)RT}\frac{\gamma_{S}}{2r}$$

where α is a geometric factor of the nucleus (e.g. α = 3 for a sphere), r the radius of a spherical particle, V the molar volume (m^3^/mol), R the ideal gas constant (J/K/mol), T the temperature (K), and γ_S _the mean surface free energy (surface tension in J/m^2^).

The surface tension γ_S _can be regarded as the total surface energy divided by the total surface area of the crystal grain. Although this relationship has only been established for colloidal particles, it is assumed that it also holds for nano-particles. The radius of the particles and the associated size-dependent term of the solubility depend in turn on the procedure followed to synthesize the solid (e.g. on aging time, impurities, etc.). In a suspension containing As-rich ferrihydrite, the redox potential (in mV) imposed by the Fe(III)/Fe(II) couple between pH 5 and 9 is given by:

$$E_{H}(Fe)=59\times \left(\log(*K_{so}K_{red})-3pH-\log a_{Fe^{2+}}\right)$$

where *K_so _is the solubility of As-rich ferrihydrite.

The redox potential imposed by the As(V)/As(III) couple between pH 5 and 6.96 is similarly given by the equilibrium between H_2_AsO_4_^- ^and H_3_AsO_3_:

$$\begin{array}{cc} E_{H}(As)=59\times \left(\frac{1}{2}\log K_{1}-\frac{3}{2}pH+\frac{1}{2}\log(\frac{a_{H_{2}AsO_{4}^{-}}}{a_{H_{3}AsO_{3}}})\right) & \text{if pH}<6.96 \end{array}$$

where K_1 _(= 10^21.76 ^at 25°C) is the reduction reaction constant of H_2_AsO_4_^- ^to H_3_AsO_3_.

At pH above 6.96, the same redox potential is given by the equilibrium between HAsO_4_^2- ^and H_3_AsO_3_:

$$\begin{array}{cc} E_{H}(As)=59\times \left(\frac{1}{2}\log K_{2}-2pH+\frac{1}{2}\log(\frac{a_{HAsO_{4}^{2-}}}{a_{H_{3}AsO_{3}}})\right) & \text{if pH}>6.96 \end{array}$$

where K_2 _(= 10^28.7 ^at 25°C) is the reduction reaction constant of HAsO_4_^2- ^into H_3_AsO_3_.

The threshold Fe^2+ ^activity (called Fe_cr_) for which E_H_(Fe) and E_H_(As) are equal, is given between pH 5 and pH 9 by:

$$\begin{array}{cc} \log Fe_{cr}=\log\frac{K_{red}*K_{so}}{K_{1}^{0.5}}-\frac{3}{2}pH-\frac{1}{2}\log(\frac{a_{H_{2}AsO_{4}^{-}}}{a_{H_{3}AsO_{3}}}) & \text{if pH}<6.96 \end{array}$$

$$\begin{array}{cc} \log Fe_{cr}=\log\frac{K_{red}*K_{so}}{K_{2}^{0.5}}-pH-\frac{1}{2}\log(\frac{a_{HAsO_{4}^{2-}}}{a_{H_{3}AsO_{3}}}) & \text{if pH}>6.96 \end{array}$$

Assuming that the redox reactions proceed under kinetic constraints due to microbial activities rather than thermodynamic equilibrium, the successive use of terminal electron acceptors can be rationalized in terms of the redox reaction energy yields [24]. When initial E_H_(As) is lower than initial E_H_(Fe) then As-rich ferrihydrite is initially more susceptible to bacterial reduction than aqueous As(V) [12]. The bacterial reductive dissolution of ferrihydrite could first occur according to the reaction:

0.125CH_3_COO^- ^+ Fe(OH)_3_(s) + 1.875H^+ ^→ 0.25HCO_3_^- ^+ Fe^++ ^+ 2.5H_2_O

at a rate R_1 _given by the law:

$$\begin{array}{cc} R_{1}=\nu_{\max}N\frac{\left[Fe(III)\right]}{K_{m}+\left[Fe(III)\right]}RPF & RPF=1-\frac{a_{Fe^{2+}}}{Fe_{cr}} \end{array}$$

where ν_max _is the maximum specific Fe(III) reduction rate per cell, N the cell density for the biomass 1 in cells mL^-1^, K_m _the half saturation constant in mM, [Fe(III)] the substrate concentration in mM, and RPF the Redox Potential Factor with Fe_cr _the maximum value of Fe^2+^(aq) activity calculated by Eq. (8) or (9).

The first term in the equation (11) predicts that DIRB will continue to metabolize until the concentration of the Fe(III) substrate vanishes (Monod empirical law). The second term of this rate law (RPF) gives an energetic explanation for a threshold phenomenon [25,26]: when the RPF is lower than a threshold value (e.g. RPF = 0.1), the bacterial reduction of As(V) in solution could also occur according to the redox reaction:

$$\begin{array}{cc} 0.{\text{25CH}}_{3}{\text{COO}}^{-}+{\text{H}}_{2}{\text{AsO}}_{4}^{-}+0.75{\text{H}}^{+}\to 0.5{\text{HCO}}_{3}^{-}+{\text{H}}_{3}{\text{AsO}}_{3}^{\text{°}} & \text{if pH}<6.96\\ 0.25{\text{CH}}_{3}{\text{COO}}^{-}+{\text{HAsO}}_{4}^{2-}+1.75{\text{H}}^{+}\to 0.5{\text{HCO}}_{3}^{-}+{\text{H}}_{3}{\text{AsO}}_{3}^{\text{°}} & \text{if pH}>6.96 \end{array}$$

at a rate R_2 _which is equal to zero if RPF > 0.1 and which otherwise is given by:

$$\begin{array}{cc} \begin{array}{cc} R_{2}=\nu_{\max}^{*}N^{*}\frac{\left(H_{2}AsO_{4}^{-}\right)}{K_{m}^{*}+(H_{2}AsO_{4}^{-})}RPF^{*} & \text{if pH}<6.96\\ R_{2}=\nu_{\max}^{*}N^{*}\frac{\left(HAsO_{4}^{2-}\right)}{K_{m}^{*}+(HAsO_{4}^{2-})}RPF^{*} & \text{if pH}<6.96 \end{array} & RPF^{*}=(1-\frac{a_{H_{3}AsO_{3}}}{As_{\max}}) \end{array}$$

where ν*_max _is the maximum specific As(V) reduction rate per cell, N* is the cell density for the biomass 2 in cells mL^-1^, K*_m _is the half saturation in mM, (H_2_AsO_4_^-^) and (HasO_4_^2-^) are the concentrations of As(V) in mM, and RPF* is the Redox Potential Factor with As_max _a maximum activity of H_3_AsO_3_.

It should be noted here that bacterial reaction in the model is assumed to proceed only in the forward direction; the case in which aqueous Fe(II) decreases after an initial increase, which should stop As(V) reduction (RPF > 0.1), is not be considered.

Interactions between aqueous species and the solid are described by a two-layer surface complexation model [27] with two types of adsorption site being considered, ≡(s)FeOH and ≡(w)FeOH for strong and weak sites, respectively. The two sites are assumed to have equal acid-base intrinsic constants and different affinities for sorbate ions (Table 2). Recommended site densities used for HFO, which has a surface area of about 600 m^2^g^-1^, are 0.005 mol/mol Fe for ≡(s)FeOH and 0.2 mol/mol Fe for ≡(w)FeOH [27]. These densities are based on maximum sorption densities, although there are substantial discrepancies in estimates of the maximum sorption density on HFO for arsenic. Adsorption maxima of 0.31, 0.4 and 0.6 mol As (mol Fe)^-1 ^have been achieved for As(III), and 0.25 mol As (mol Fe)^-1 ^for As(V) [28,29]. It should be noted that Dixit and Hering [30] recently observed an increase of 0.4 mol As(III) per mol Fe(II) adsorbed. Here we used a value of 0.4 mol/mol Fe for weak sites concentration in the modeling of our high As/Fe molar ratio experiments and 0.2 mol/mol Fe in the modeling of Bengali sediments experiments with a lower As/Fe molar ratio. This sorption site concentration decreases linearly with the solid Fe concentration.

**Table 2:** Complexation reactions with the constants used in the model

Aqueous and surface reactions	log K (I = 0 M, 25°C)	Reference
H_3_AsO_4_° = H_2_AsO_4_^- ^+ H^+^	-2.25	[32]
H_2_AsO_4_^- ^= HAsO_4_^2- ^+ H^+^	-6.96	[32]
HAsO_4_^2- ^= AsO_4_^3- ^+ H^+^	-11.5	[32]
FeH_2_AsO_4_^+ ^= H_2_AsO_4_^- ^+ Fe^2+^	-2.86	[32]
FeHAsO_4_° + H^+ ^= H_2_AsO_4_^- ^+ Fe^2+^	3.42	[32]
FeAsO_4_^- ^+ 2H^+ ^= H_2_AsO_4_^- ^+ Fe^2+^	11.04	[32]
H_3_AsO_3_° = H_2_AsO_3_^- ^+ H^+^	-9.22	[33]
H_2_AsO_3_^- ^= HAsO_3_^2- ^+ H^+^	-12.11	[33]
HAsO_3_^2- ^= AsO_3_^3- ^+ H^+^	-13.41	[33]
≡(w)FeOH + H^+ ^= ≡(w)FeOH_2_^+^	7.29	[27]
≡(w)FeOH = ≡(w)FeO^- ^+ H^+^	-8.93	[27]
≡(s)FeOH + H^+ ^= ≡(s)FeOH_2_^+^	7.29	[27]
≡(s)FeOH = ≡(s)FeO^- ^+ H^+^	-8.93	[27]
≡(w)FeOH + AsO_4_^3- ^+ 3H^+ ^= ≡(w)FeH_2_AsO_4 _+ H_2_O	29.88	[28]
≡(w)FeOH + AsO_4_^3- ^+ 2H^+ ^= ≡(w)FeHAsO_4_^- ^+ H_2_O	24.43	[28]
≡(w)FeOH + AsO_4_^3- ^+ H^+ ^= ≡(w)FeAsO_4_^2- ^+ H_2_O	18.10	[28]
≡(w)FeOH + AsO_3_^3- ^+ 3H^+ ^= ≡(w)FeH_2_AsO_3 _+ H_2_O	38.76	[28]
≡(w)FeOH + AsO_3_^3- ^+ 2H^+ ^= ≡(w)FeHAsO_3_^- ^+ H_2_O	31.87	[28]
≡(w)FeOH + CO_3_^2- ^+ H^+ ^= ≡(w)FeOCO_2_^- ^+ H_2_O	12.78	[34]
≡(w)FeOH + CO_3_^2- ^+ 2H^+ ^= ≡(w)FeOCO_2_H + H_2_O	20.37	[34]
≡(w)FeOH + Fe^2+ ^= ≡(w)FeOFe^+ ^+ H^+^	-2.98	[34]
≡(s)FeOH + Fe^2+ ^= ≡(s)FeOFe^+ ^+ H^+^	-0.95	[34]

Modeling geochemical speciation, sorption and microbial reactions was done with the program REACT included in the Geochemist's Workbench^® ^Release 4.0.3 [31]. Activity coefficients in REACT were calculated using the Debye Hückel law (at an ionic strength of about 0.04 M). Aqueous reaction constants used for thermodynamic calculations are the values included in the LLNL version 8, release 6 dataset (called "thermo.com.V8.R6+.dat" by REACT), except for some arsenate and arsenite complexes listed in Table 2. The protonation and ferrous complexation constants of arsenate were recommended by Whiting (1992) as a result of a literature research [32]. The protonation constants of arsenite, being consistent with the sorption data calculated by Dixit and Hering [28], were extracted from the MINEQL V4.5 database [33]. The equilibrium sorption constants used in REACT are based on data reported by Dzombak and Morel (1990) [27], apart from more recent data for arsenate As(V) and arsenite As(III) [28] and for ferrous iron Fe(II) and carbonate species [34], which are listed in Table 2.

## Experimental results

### Iron speciation

**Figure 1:**
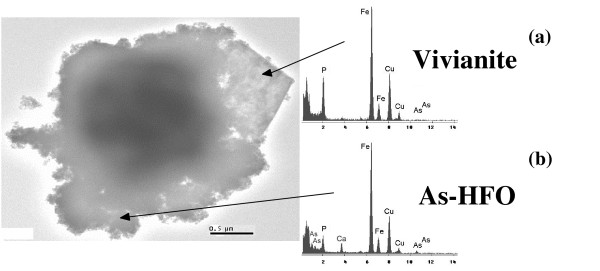
TEM image in CP1 experiment with FR after 2 months. Scale bar = 0.5 μm. EDS insets indicate two kinds of Fe-minerals: (a) Vivianite crystal with high amounts of Fe and P, (b) 2LFh containing minor amounts of As, P and Ca.

The XRD diagrams show that As-2LFh synthesized in the laboratory corresponds to 2-line ferrihydrite, clearly characterized by two diffuse bands at 2.55 and 1.45 Å. The As-2LFh sterilization step in the culture medium at 100 °C had no measurable effect on the crystallinity of the solid. Moreover, most of the solids after two months of incubation were still 2-line ferrihydrite. Vivianite Fe(II)_3_(PO_4_)_2_.8H_2_O was also clearly identified by X-ray diffraction analysis, but to a lesser extent and only after the second month of experiments AD1 and CP1 with FR (Table 1). A close association between vivianite and 2-line ferrihydrite in experiment CP1 with FR was noted from Scanning Transmission Electron Microscopy observations (Fig. 1). No green-rust or magnetite was identified, but bobierrite Mg_3_(PO_4_)_2_.8H_2_O was characterized in experiment AD1.

**Figure 2:**
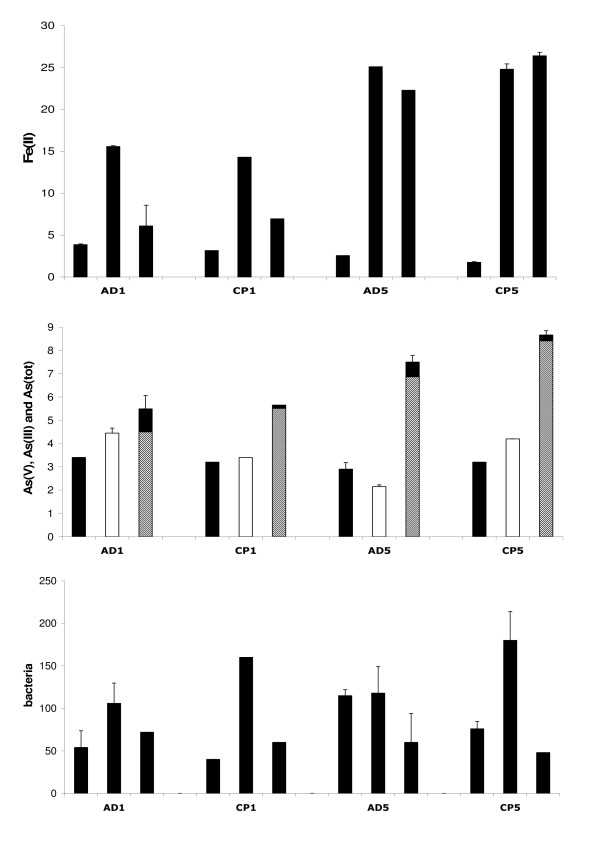
Concentrations of aqueous Fe(II) in mg/L, of aqueous As(V) (black bars), As(III) (gray bars) and As(tot) (white bars) in mg/L, and of bacteria in million cells/mL. Each group of 3 bars includes data of experiments AD1, CP1, AD5, CP5 after 1 day, 28 days and 63 days of As-2LFh incubation. Error bars represent the standard deviation of duplicated measurements.

Iron concentrations in the aqueous phase were monitored during the incubations under strict anaerobiosis at initial pH ~6, both with and without inoculation of FR. Because FR cell suspensions were maintained with Fe(III) as growth electron acceptor (See Additional File 1), the inoculum was not free of Fe(II); consequently there was an initial concentration of aqueous Fe(II) in the incubation experiments with FR bacteria (Fig. 2). In addition, ferrous iron Fe(II) was the predominant valence state of iron at the beginning of incubation in the experiments with FR.

**Figure 3:**
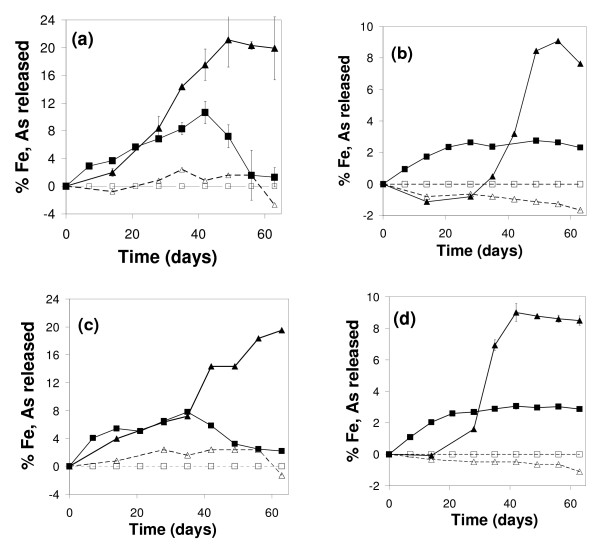
Aqueous Fe (squares) and As (triangles) concentrations released during experiments AD1 (a), AD5 (b), CP1 (c), CP5 (d). Empty symbols represent control experiments without bacteria. Proportions of released Fe and As are calculated by subtracting the concentration at the beginning of each experiment (Fig. 2) from the concentrations measured over time, and then dividing the differences by the initial solid content of Fe and As (Table 1), respectively. Error bars represent the standard deviation of duplicated measurements.

**Figure 4:**
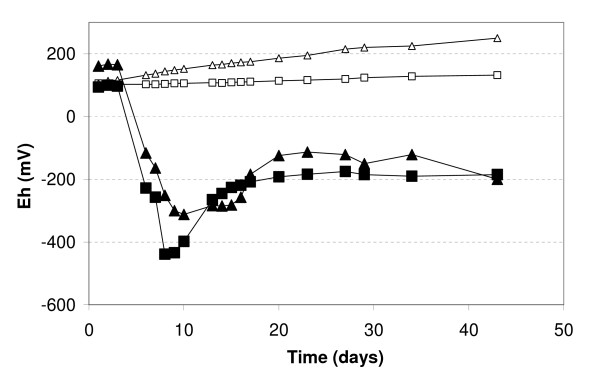
Eh recorded during experiments CP1 (squares) and CP5 (triangles). Empty symbols represent control experiments without bacteria.

Fe release was observed only in samples inoculated with FR, and not in the control experiments (Fig. 3). Moreover, the kinetics of Fe(III) reductive dissolution occurred in two phases during the two-month period of incubation: a first phase of increasing dissolved iron concentration Fe(II) during the first month (Figs. 2 and 3), associated with bacterial growth (Fig. 2) and a decrease of Eh (Fig. 4); a second phase, during the second month, marked by a stabilization of aqueous Fe concentrations in experiments AD5 and CP5 and even by a decrease in experiments AD1 and CP1. The maximum Fe release was about 3% in experiments CP5 and AD5 and 8–10% in experiments CP1 and AD1 (Fig. 3). The initial reduction rate was higher in the CP than the AD experiments (Table 3), but there was no difference in the percentage of released Fe at the end of the experiments (Fig. 3).

**Table 3:** Experimental conditions and initial rates of bacterial reductive dissolution of As-2LFh compared to As-free 2LFh experiment and literature data.

	AD1	CP1	AD5	CP5	2LFh	HFO^a^
Bacteria	FR	FR	FR	FR	FR	*Shewanella putrefaciens*
Initial cell density (10^6 ^cells/mL)	54	40	11	76	no data	100
Initial R_1 _(10^-7 ^mMs^-1^)	1.5	2	2.4	2.7	17.2	18.6

^a ^Taken from [43]

### Arsenic speciation

Analytical results indicate that about the same concentration of As was solubilized in pentavalent form within few hours after each incubation with and without FR. This initial solubilization of about 3 mg L^-1 ^(~40 μM in Table 4), which represents about 5% of the total As quantity for experiments AD5 and CP5 and 25% for AD1 and CP1, seems not to depend on the initial solid concentration of As-2LFh (Fig. 2). So as to distinguish between abiotic and biotic release of As, and also to facilitate comparison between As and Fe release, the proportions of As and Fe release have been calculated in Figure 3 by subtracting the As and Fe concentrations after initial equilibrium from the concentrations measured over time, and normalizing the differences to the total quantity of As and Fe (Table 1).

**Table 4:** Final aqueous concentrations after 63 days for the CP5 incubation experiment with and without (control) FR bacteria.

Aqueous concentrations	With FR bacteria	Without bacteria
As (μM)	120	40
molar As(III)/As (%)	97	0
Fe (μM)	466	36
PO_4 _(μM)	153	252
Ca (μM)	735	638
Mg (μM)	506	477
Na (mM)	41.3	40
NH_4 _(mM)	2.8	3.9
HCO_3_^- ^(mM)	18.7	12.3
Acetate (mM)	8.5	18.6

During the first month of experiments AD1 and CP1 with FR, the release of arsenic seemed to be congruent with the release of iron, i.e. about the same proportion of Fe and As was released (Fig. 3). Conversely, there was no congruent release of arsenic during the first month of incubation with experiment CP5 with FR, and even a small decrease of aqueous As concentration in experiment AD5.

For the second month of incubation with FR, no difference was observed between the CP and AD experiments with respect to the final percentage of As released. About 20% of arsenic was released in experiments AD1 and CP1 and about 9% in experiments AD5 and CP5 (Fig. 3).

At the end of the two-month incubation with FR, the analytical results showed that more than 90% of the aqueous As was in the trivalent form As(III) (Fig. 2).

**Figure 5:**
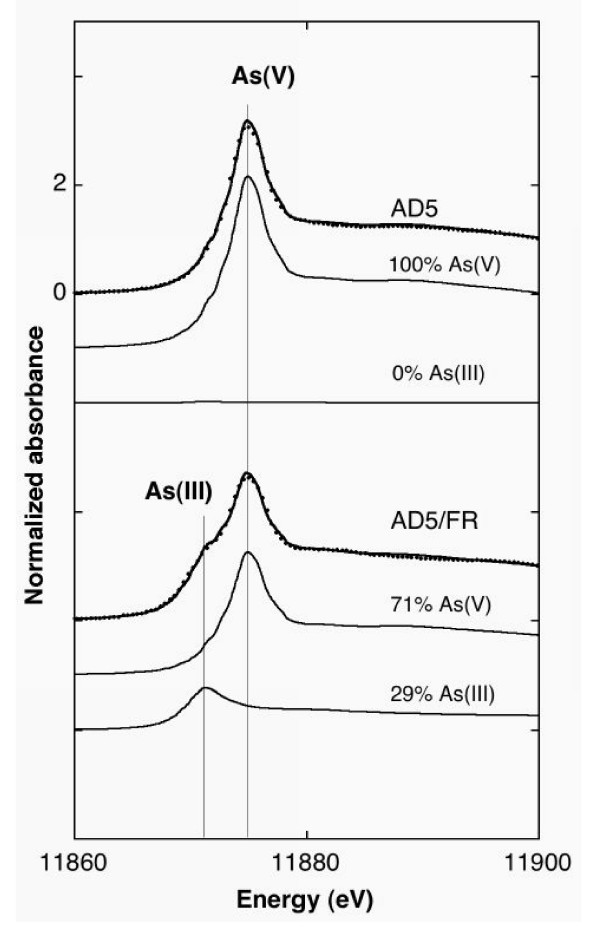
As K-edge XANES spectra after 63 days. Abiotic (AD5), biotic with FR (AD5/FR). Dots: experimental; Plain line: linear least-squares fit. Weighted spectra of the fitting components (see text) are plotted as plain lines below each fitted spectrum.

The oxidation state of sorbed and co-precipitated As was measured directly by XANES (Fig. 5). Before incubation and also after abiotic incubation, the solid As was only in its pentavalent form As(V). After 21 days of incubation with FR, X-ray absorption analysis indicated that the solid phase was still As(V) dominant (>70%) (Table 1).

### Isolation of a single arsenate-reducing strain from the FR

The tests conducted in the selective medium for dissimilatory arsenic-reducing bacteria (DARB) described by Kuai et al. [35] clearly show that at least one strain within the FR community was able to respire As(V). This strain, FRB1 which respires both Fe(III) and As(V), was isolated from the FR community and characterized by molecular biology tools. Supplemental data supporting the given assignments are available in Additional File 1.

### Incubation experiments with Bengali sediments (Islam et al., 2004)

The biogeochemical regime described for the incubation of Bengali sediments by Islam et al. (2004) is very close to our experiments [12]. The observed release of arsenic during incubation with an amendment of 4 gL^-1 ^sodium acetate seemed to follow exactly the same pathway as in our As-2LFh experiments: a limited release of Fe(II) during the first 20 days followed by an arsenic release over about 10 days, and a co-existence in the final state of a dominant As(V) solid phase and a dominant As(III) aqueous phase.

## Discussion and modeling

### Iron reductive dissolution

#### Transformation of solid iron

Amorphous ferrihydrite is known to transform rapidly to more crystalline minerals with temperature [36] and/or time [16]. Several authors, however, have shown that the evolution of 2LFh crystallinity with time is largely reduced or inhibited in the presence of namely arsenic [37] or phosphate [38]. The inhibiting role of high arsenic or phosphate contents for crystallization could therefore explain why most of the solid phase detected by XRD remained as As-2LFh after the heat sterilization step. The detection limit of the XRD method is around 1–5 wt.%, depending namely on the mineral's degree of crystallinity and symmetry. Thus XRD identification of new, poorly crystallized phases present in minor amounts is difficult.

**Figure 6:**
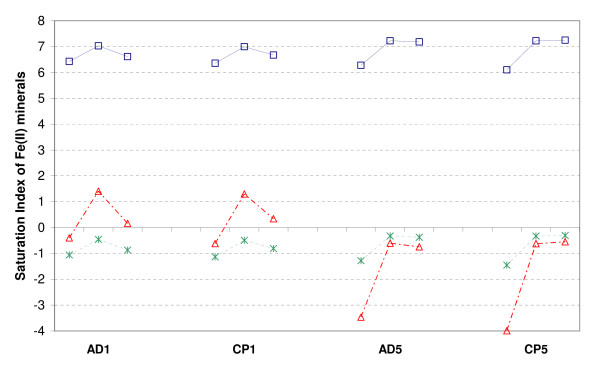
Saturation index of the Fe(II) minerals siderite (stars), vivianite (triangles) and magnetite (squares). Each group of 3 points includes data of experiments AD1, CP1, AD5, CP5 after 1 day, 28 days and 63 days of As-2LFh incubation, assuming pH = 6.

In experiments AD1 and CP1, a kinetic precipitation of some ferrous phase such as siderite, magnetite or vivianite [39,40] could explain the dramatic drop in soluble iron that appears during the second month (Figs. 2 and 3). The scenario of a redox reaction involving aqueous As(V) reduction by aqueous Fe(II) is rejected because this reaction is known to be kinetically limited at circumneutral pH [41]. An equilibrium modeling using the experimental data of aqueous Fe(II) after 1 day, 28 days and 63 days and the logK listed in Table 5 suggests that the solution was always undersaturated with respect to siderite and always oversaturated with respect to magnetite (Fig. 6). In fact neither siderite nor magnetite were detected by XRD in our experiments. The logK of magnetite is perhaps not well defined and underestimated due to the nano-size of the amorphous precursors. Alternatively, the XRD detection limit was perhaps not low enough to detect the formation of this phase in small amounts and/or in amorphous form. The solution was undersaturated with respect to vivianite Fe(II)_3_(PO_4_)_2_.8H_2_O, at the beginning of experiments AD1 and CP1, oversaturated after 28 days, and not far from equilibrium after 63 days (Fig. 6). This modeling result is consistent with the XRD detection of vivianite after 63 days in experiments AD1 and CP1 (Table 1), and with the hypothesized process of a kinetic precipitation of vivianite in these experiments.

**Table 5:** Iron(II,III) minerals and the solubility constants used in the model

Reactions	Log K_so _(I = 0 M, 25°C)	Reference
Fe(OH)_3_(s) + 3 H^+ ^= 3 H_2_O + Fe^3+^	5.66	(1)
As-2LFh + 3 H^+ ^= 3 H_2_O + Fe^3+^	5.6	From Eq. (4)
Siderite + H^+ ^= Fe^2+ ^+ HCO_3_^-^	-0.19	(1)
Magnetite + 8 H^+ ^= Fe^2+ ^+ 4 H_2_O + 2 Fe^3+^	10.47	(1)
Vivianite + 2 H^+ ^= 3 Fe^2+ ^+ 8 H_2_O + 2 HPO_4_^2-^	-11.07	[62]

(1) Taken from LLNL version 8, release 6 dataset (thermo.com.V8.R6+.dat)

No secondary phase was detected by XRD in experiments AD5 and CP5 (Table 1) and the equilibrium model shows that the solution was always undersaturated with respect to vivianite (Fig. 6) because of a higher available sorption surface for phosphates.

Recent results from Root at al. [42] on arsenic sequestration in high-iron sediments show from X-ray Absorption Spectroscopy (XAS) that "the HFO floc had been reduced to a mixed Fe(II,III) solid with a local structure similar to that of synthetic green rust (GR)", but also show that there is no evidence from XRD for the formation of a crystalline GR phase.

The Fourier transform infrared spectrometry (FTIR) technique was used in conjunction with XRD to identify the presence of other slightly crystalline phases. Spectra of the raw products were recorded using a Bruker Equinox IFS55 spectrometer with a spectral resolution of 4 cm^-1^, and the attenuated total reflectance (ATR) mode was adopted directly on the powder with a 4000–550 cm^-1 ^range. The FTIR shows the presence of 2LFh and of sorbed species such as phosphates and arsenic already identified by energy-dispersive X-ray analysis (EDS), but does not show the presence of any new Fe- and/or As-bearing phases.

#### Dissimilative or assimilative iron reduction

Control experiments without FR (Fig. 3) show that the observed ferric iron reduction was due to a biological mechanism, which could be a priori either dissimilative or assimilative. The residual glucose present in the inoculum was measured at the beginning of each experiment: a maximum glucose value of 10 mg/L was found in the CasR1 medium, whilst 10 g/L were initially in the Bromfield medium used for FR maintenance (See Additional File 1). Consequently the main carbon sources in the experiments were not glucose (a well-known fermentable carbon source), but acetate and lactate, which are commonly used by DIRB. Moreover, the decrease of acetate concentration after 2 months (Table 4) would not be observed if fermenting bacteria were present at much higher abundance than DIRB in the FR community.

The values of initial aqueous Fe(II) production rate are calculated for AD1, CP1, AD5 and CP5 experiments with FR and compared to the maximum dissimilatory reduction rate ν_max_N of HFO by *S. putrefaciens *reported by Bonneville et al. (2004) [43] (Table 3). The observed rates in all experiments are one order lower than the As-free HFO dissimilatory reduction rate. Moreover, this initial rate does not explain the observed evolution of Fe release with time, i.e. why the As-2LFh was incompletely reduced in all the experiments (between 3 and 10% release of Fe in Fig. 3). A similar limited release has been reported in batch studies with metal-substituted goethite [44], and a greater Fe(II)/Fe_tot _ratio of 40 to 60% was achieved by reduction of HFO by *S. putrefaciens *CN32 [39].

**Figure 7:**
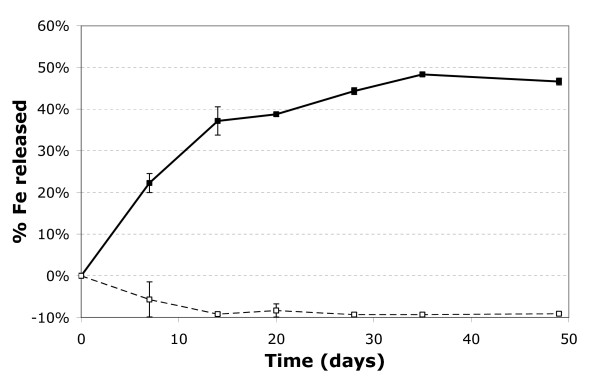
Aqueous Fe over time during As-free 2LFh experiment (full line with filled squares). Empty symbols are used for the control experiment without bacteria. Proportion of Fe released is calculated by subtracting the concentration at the beginning from the concentrations measured over time and by dividing these differences by the initial solid content of Fe. Error bars represent standard deviation of duplicated measurements.

In order to clarify this important point, additional reduction experiments were carried out with 2LFh, but without As, in order to study the specific role of arsenic. The results (Fig. 7) show that the FR population was able to reduce about 50% of the 2LFH, like other known DIRB such as *S. putrefaciens *with a similar initial rate (Table 3).

DIRB were therefore probably also present in high abundance in the experiments with As-2LFh, with arsenic directly or indirectly playing an inhibiting role during the dissimilative iron reduction of As-2LFh. The inhibiting role of arsenic in As-2LFh experiments is discussed in more detail in the next section.

### Arsenic release

#### Initial ion exchange with phosphates

A similar initial solubilization of arsenic occurred both in the experiments with FR and in the control experiments without FR (data not shown). This result indicates that a chemical, and not a biotic, mechanism led to an initial release of As during the equilibrium of the solid As-2LFh with the growth medium. Additional experiments then showed that the initial release of As(V) depends on the initial concentration of phosphates. In fact, it was already known that phosphates in growth media have a high affinity for iron-oxide surfaces and can compete with arsenate for adsorption sites [45]. For the sorption of the different oxyanions onto As-2LFh during experiments AD5 and CP5, we used the surface complexation model of Dzombak and Morel (1990) [27] with the intrinsic surface complexation constants given in Table 2. The model, set with a pH of 6.3, correctly simulated the initial aqueous concentration of As(V) corresponding to 5% of total As, i.e. a sorbed fraction of 95%.

#### Congruent dissolution with iron

After the initial chemical release of As, the release during the first month depended on the initial solid concentration of As-2LFh.

In experiments AD1 and CP1 with FR, the proportion of As release was very similar to the proportion of Fe release (Fig. 3). Bousserrhine (1999) [46,47] similarly shows that the release of co-precipitated metals like Co or Mn from goethite is congruent with that of Fe(II); a result consistent with the hypothesis that As(V) was uniformly distributed within the co-precipitate. This is a result that was expected in experiment CP1, but not in experiment AD1 where As was added to a preformed 2LFh precipitate. Both samples showed a similar molar ratio (about 5.5% in Table 1), so perhaps some early transformation could explain a uniform distribution of the As during the preparation of the AD1 sample.

Conversely, there was no congruent release of As during this first month of incubation with FR during experiment CP5, and there was even a small decrease during experiment AD5 (Fig. 3). This could mean that the re-sorption of released As(V) in experiments CP5 and AD5 was higher because of the larger available sorption surface. It should be noted that Pedersen et al. (2006) [48] have recently shown that a congruent release of arsenic and iron during abiotic reductive dissolution of iron oxides cannot be expected at an As/Fe molar ratio of less than 0.5%.

#### Bacterial reduction

As there was no measured aqueous As(III) in the control experiments, an indirect or direct bacterial activity must have been the cause of As(V) reduction. As(V) reduction by aqueous Fe(II) or adsorbed Fe(II) produced by biotic As-2LFh dissolution would be an indirect biotic mechanism. Note that we have rejected the scenario of a redox reaction involving aqueous As(V) reduction by aqueous Fe(II) because this reaction is known to be kinetically limited at circumneutral pH [41]. Charlet et al. (2002) [49] suggest another abiotic mechanism that was observed in the reduction of U(VI) by surface Fe(II) [50] – the reduction of As(V) coupled to the oxidation of adsorbed Fe(II). Although this indirect mechanism cannot be excluded, a direct biotic As(V) reduction would be also consistent with the observation that dissimilatory As(V)-reducing bacteria were present in the FR community. The incubation of FR in a selective medium for DARB did indeed show the presence of such microorganisms within the FR [35]. One strain (FRB1) isolated from the FR belongs to the genus *Clostridium *which is able to respire both electron acceptors, Fe(III) and As(V) (see Additional File 1).

As(V) is used as a terminal electron acceptor by several phylogenetically diverse bacteria, and there are recent reports on the implication of *Clostridium *species in As(V)-reducing activity: e.g. sp. OhilAs (Oremland and Stolz, 2003), ARCL1 and AKAR3 (Rhine et al., 2005) used As(V) as a respiratory electron acceptor. However, the 16S RNA sequences of these species were not available in databases and could not be compared to the sequence of strain FRB1 (See Additional File 1). Such comparison is necessary to know whether strain FRB1 is related to these *Clostridium *microorganisms. Nevertheless, despite its close phylogenetic relationship with *C. pasteurianum *on 16S rRNA genes and because of its ability to reduce As(V), strain FRB1 could represent a novel species of the genus *Clostridium*. Although Islam et al. (2004) [12] recently retrieved sequences related to *Clostridium *species from sediments in West Bengal, there is no evidence that these organisms are also As(V)-reducing bacteria.

The recent work of Campbell et al. (2006) [51] addresses the relative order of microbial As(V) and Fe(III) reduction, as well as the effect of sorbed As(V) and As(III) on rates of Fe reduction. Synthetic iron oxyhydroxides HFO were incubated with two types of microbial inoculum: one a microbial community from the Haiwee reservoir and the other a well-studied laboratory strain, *Shewanella *sp. strain ANA-3 wild type (WT), which is capable of both Fe(III) and As(V) reduction. In all the experiments, As(V) was reduced simultaneously with or prior to Fe(III), and the As(V) adsorbed onto the surface of HFO enhanced the rate of microbial Fe(III) reduction by the pure strain ANA-3 WT, i.e. the exact opposite of our experiments. Note, however, that our FR community a) was not selected from an As-impacted field site like the Haiwee reservoir, but from a site contaminated by heavy metals, and b) was obtained by enrichment of Fe(III)-reducing bacteria. In our opinion, the Haiwee bacterial community has a much lower DIRB than DARB abundance, whereas the opposite is probable in our FR community.

### Decoupling of iron and arsenic release

Based on the results described in the previous sections, and in agreement with Islam et al. (2004), we suggest that solid Fe(III) and As(V) will be used by the biomass sequentially and we show that the sequence can be explained by the energy yield of both reduction reactions. This yield depends on redox potentials: at the beginning of bacterial growth, As-rich ferrihydrite reduction is energetically more favorable than arsenic reduction, but with increasing Fe(II) concentration, a point is reached where aqueous As(V) reduction becomes an alternative for the FR community bacteria, which can respire both As(V) and Fe(III). Our biogeochemical model assumes that the biomass growth is in two parts in Eq (11) and (13): biomass 1 with only DIRB species, and a initial minor amount of biomass 2 with species like FRB1 that can respire both As(V) and Fe(III). In the mathematical modeling, therefore, given the potential energy changes, the more efficient growth microbes (biomass 2) suppress the less efficient growth microbes (biomass 1).

This energetically driven model was tested both on our own results and on those obtained from the incubation experiments with Bengal delta sediments [12].

#### The As-ferrihydrite system

In the As-ferrihydrite experiments run in the present study, the Fe(III)/Fe(II) redox potential Eh in mV is given by Eq. (5). Thus the more Fe(III) that is released in solution and the more soluble the solid phase, the higher the Eh. The solubility of synthetic pure ferrihydrite reported in the literature varies over two orders of magnitude with logK_so _ranging from 4.3 [52] to 2.5 [53]. The highest value was measured for fresh precipitates and the minimum value for partially crystallized particles. It has been shown that oxyanions, like arsenate, silicate and phosphate, poison the growth of precursor particles during the course of ferrihydrite precipitation [37,54,55]. Therefore, As-rich ferrihydrite can be expected to have a greater solubility than As-free ferrihydrite. The particle size of ferrihydrite was found to be 8–15 Å in the initial stages of polymerization [37]. The solubility of 15 Å-diameter particles of amorphous As-rich ferrihydrite is estimated by a value of log*K_so _= 5.6 using Eq. (4), with a interfacial tension γ_S _= 1.6 J/m^2 ^[56], a solubility logK_sp _= -2.1 for a well-crystallized goethite [57], and a molar volume V = 20.6 cm^3 ^mol^-^^1^. It should be noted that the resultant log*K_so _is equal to the solubility of As-free Fe(OH)_3 _(logK = 5.66) given in the LLNL V8 R6 thermodynamic database (Table 5). This reveals the large uncertainty in the log*K_so _of 2-line ferrihydrite and As-2LFh.

Assuming this value of log*K_so _and an initial Fe^2+^(aq) activity of 4.10^-6 ^corresponding to an initial concentration of 1.76 mg/L of Fe(II), an E_H_(Fe) of about 300 mV is calculated using Eq. (5) at pH 6.3. Then assuming an initial As(V) dominant aqueous phase with an As(V)/As(III) activity ratio between 10 and 10^5^, the range of E_H_(As) is calculated, using Eq. (6), as +112 to +230 mV, in all cases lower than the initial E_H_(Fe). Therefore, As-rich ferrihydrite is initially more susceptible to bacterial reduction than aqueous As(V).

**Figure 8:**
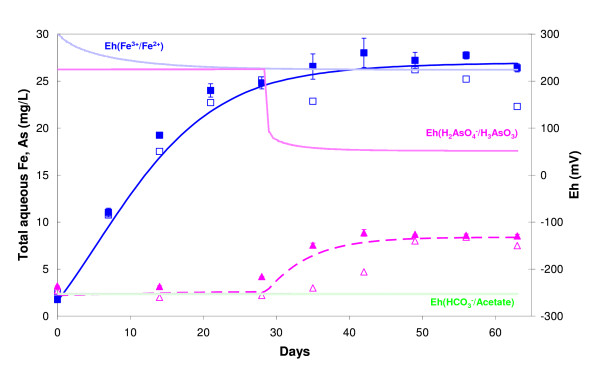
Comparison between experimental data and REACT model: aqueous Fe (squares) and As (triangles) concentrations over time during CP5 (filled symbols) and AD5 (empty symbols) experiments. Monod kinetics was used to model Fe (filled line) and As (dotted line). Eh was computed by the model from simulated redox couples Fe^3+^/Fe^2+^, H_2_AsO_4_^-^/H_3_AsO_3_°, HCO_3_^-^/Acetate.

The selected values in Eq. (11) for K_m_, ν_max_N and Fe_cr _and in Eq. (13) for K*_m_, ν*_max_N* and As_max _are listed in Table 6. The initial As(III) concentration is under the detection limit (about 5 μg L^-1^) and, therefore, the critical Fe^2+ ^activity (Fe_cr_) can not be deduced from Eq. (8). The value of Fe_cr _was fitted using the observed maximum Fe(II) concentration (25 mg L^-1^). Afterwards, an As(V)/As(III) activity ratio of about 6.1 10^4 ^was calculated using Eq. (8) with this value of Fe_cr_. Simulated As release is controlled in the model by the difference of Fe(III)/Fe(II) and As(V)/As(III) redox potentials: the bacterial As(V) reduction first occurred when both calculated potentials were very close (RPF = 0.1, see Fig. 8), i.e. when a_Fe_^2+ ^= 0.9 Fe_cr_. Over the 63 days of the experiment, total aqueous Fe and As concentrations were correctly simulated with these parameters for experiments AD5 and CP5 (Fig. 8). The parameters are, however, better adapted for experiment CP5 than for experiment AD5.

**Table 6:** Kinetic parameters used in the model

Parameters for Eq. (11) and (13)	Modeling of AD5 and CP5 experiment	Modeling of Islam's experiment (2004)
ν_max_N (10^-7 ^mMs^-1^)	2.74	15
K_m _(uM)	520	520
Fe_cr_* 10^3^	0.0845^a^	2.1^b^
ν*_max_N* (10^-7 ^mMs^-1^)	3.3	0.14
K*_m _(uM)	1.4	0.001
As_max_* 10^6^	92	0.15

^a ^fitted value obtained using the observed maximum Fe(II) concentration

^b ^From Eq. (9)

We also compared the final speciation of As in the aqueous and solid phases calculated by the model and experimental results. The model provided evidence of the possible co-existence of a dominant As(V) solid phase and a dominant As(III) aqueous phase: calculated As(III)/As(Total) was about 82% in the aqueous phase and about 13% in the solid phase, while the experimental values were more than 90% in the aqueous phase (Fig. 2 and Table 4) and less than 29% in the solid phase (Fig. 5 and Table 1). This result is actually not related to fitting parameters, but is constrained by the intrinsic sorption constants of Table 2 (values from the literature).

The model result for the aqueous phase was imposed by the fitting parameter As_MAX _introduced to simulate the fact that not all As(V) was bioavailable for microbial reduction. Two scenarios are proposed to explain why bacterial As(V) reduction stopped: one is a lack of phosphate availability (phosphate being is essential for the biomass growth) as suggested by the decrease in phosphate concentration in Table 4, and the other is the toxicity of As(IIII) which could inhibit cell growth.

#### The Bengali sediment system

The model was applied to the results of the experiment of [12] using the same solubility of As-rich ferrihydrite (log*K_so _= 5.6) and the same carbonate and phosphate concentrations. The main differences with the modeling of AD5 and CP5 experiments are: (1) an initial Fe solid concentration of 62.68 mM instead of 15.3 mM, (2) an initial molar ratio As/Fe in the solid of only 0.019% instead of 5.55%, (3) pH of 7 instead of 6.

The maximum activity Fe_cr _of 2.10^-3 ^was not fitted but calculated by using Eq. (9). All others parameters are fitting parameters and listed in Table 6. The initial Fe reduction rate ν_max_N from Eq. (11) was increased by about a factor of five in comparison with that used to describe the present study with As-2LFh (15 10^-10 ^instead of 2.74 10^-10 ^Ms^-1^). This value for As-poor sediment is still lower but quite close to the reduction rate of As-free 2LFh experiment and to the value found by Bonneville et al. (2004) (Table 3).

**Figure 9:**
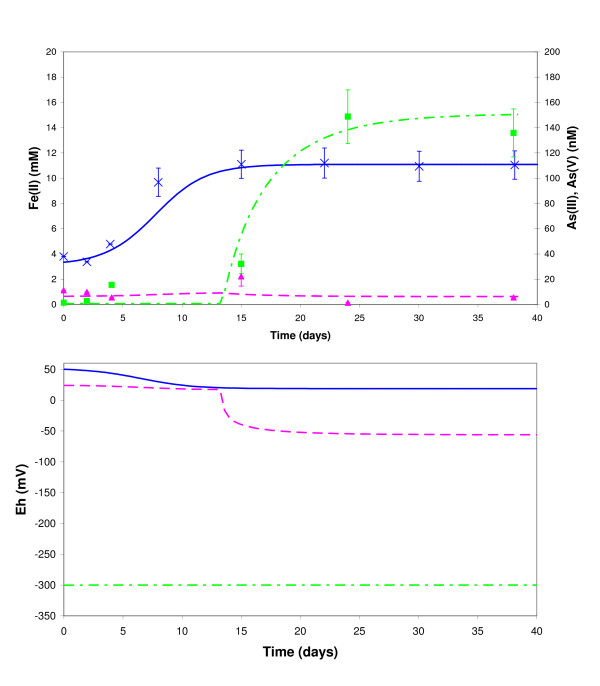
Comparison between Islam et al.'s data (2004) and REACT model: aqueous Fe (crosses), As(V) (triangles) and As(III) (squares) concentrations over time during Islam's experiment. Monod kinetics was used to model Fe (full line), As(V) (dashed line) and As(III) (dashed-dotted line). Eh was computed by the model from simulated redox couples Fe^3+^/Fe^2+ ^(full line), HAsO_4_^2-^/H_3_AsO_3_° (dashed line) and HCO_3_^-^/Acetate (dashed-dotted line).

The corresponding maximum Fe(II) concentration of 11 mM is correctly observed after about 15 days in the modeling with these parameters (Fig. 9). The biogeochemical model, initially developed on our data is therefore successfully applied to an independent dataset with natural sediments (Islam et al.). This does not, in itself, provide evidence that the sequential microbial reduction of Fe(III) and As(V) is taking place, but it does indicate that this postulated process could take place with two quite different Fe and As solid concentrations.

Islam et al. (2004) also observed the possible co-existence of a dominant As(V) solid phase and a dominant As(III) aqueous phase, as in the As-2LFh experiments. They suggest that not all the solid As(V) in Bengali sediments was bioavailable for microbial reduction "possibly through its association with recalcitrant crystalline Fe oxides". Our experiments show that this was also the case even with amorphous iron oxyhydroxides like 2-line As-rich ferrihydrite.

### Competition between As, phosphates and carbonates at 2Lfh surface sites

In the previous section, the plausibility of a sequential microbial reduction of Fe(III) and As(V) controlled by the relative energy gain of the two processes was tested with a model. We then decided to apply a model sensitivity analysis to evaluate the plausibility of other possible explanations for the decoupled release, such as: (1) a release of As induced by the release of phosphates due to the microbial reduction of iron oxides, which is a scenario described by the British Geological Survey as one of several possible mechanisms for the "*iron oxide reduction hypothesis*" [58]; and (2) a release of As induced by the competition with carbonates for surface sites [34], a scenario whereby the reduction of iron oxides and the dissolution of carbonate produce the bicarbonate that triggers the desorption of As.

**Figure 10:**
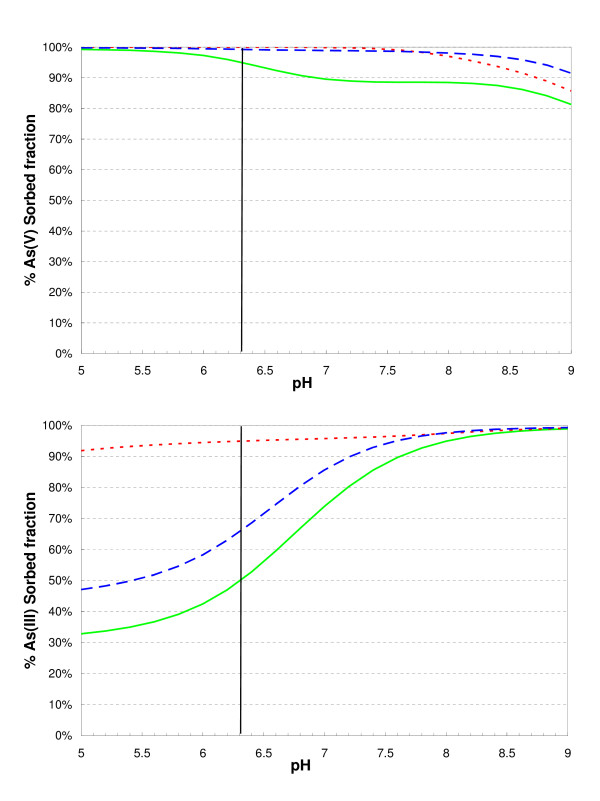
Model simulation of As(V) and As(III) sorption onto 2LFh (15.3 mM) with sorption of phosphates and carbonates (solid line) and without sorption of phosphates (dashed line) or carbonates (dotted line). Total carbonate and phosphate concentrations are 14.75 mM and 1.47 mM, respectively.

The calculated sorbed As(III) and As(V) fractions were systematically investigated with the model for a pH between 5 and 9 (Fig. 10). This sensitivity analysis showed that phosphate is a stronger competitor than carbonate for As(V) (notably in the pH 8–9 range), but that the reverse is true for As(III). In the pH 5–7 range, the calculated sorbed As(III) fraction depends strongly on the pH. For example, about 50% of As(III) was sorbed in the reference simulation at pH 6.3. Eliminating the sorption of phosphate species onto As-2LFh provided only a slight increase in the sorbed As(III) fraction (about 65% at pH 6.3), whereas by eliminating the sorption of carbonate species onto 2LFh resulted in a strong increase (about 95% at pH 6.3). ≡(w)FeOCO_2_H (Table 2) was indeed the dominant predicted sorbate. Because As(III) and carbonate species are both by-products of the bacterial redox reaction combining the reduction of As(V) and the oxidation of acetate (Eq. 12), the mobilization of arsenic could be controlled by the competition of arsenite As(III) with carbonates for the sorption sites of 2LFh. One of the above listed processes, i.e. the hypothesis of Appelo et al. (2002), could therefore account for uncorrelated release of aqueous Fe and As in both our and Islam's (2004) experiments.

This conclusion is not inconsistent with the experimental results of Radu et al. (2005) [59], who studied the effect of dissolved carbonate on As(V) and As(III) adsorption to iron-oxide-coated sand at pH 7. Radu et al. conclude that the competition effect between As(V) and dissolved carbonate was relatively small. Their result concerning the competition with As(III) was, however, more important (see expt 5, in Radu et al.).

### Alternative mechanisms omitted in the biogeochemical model

Other mechanisms that were not included in our modeling can explain some experimental observations. For example, the model did not consider the consumption of carbon and phosphorus by metabolic active microorganisms. Here, ATP production could be an explanation for the decrease in phosphate concentrations (Table 4). As for carbon, in order to test its stoichiometric molar balance, the transformed moles of acetate were compared with the produced moles of bicarbonates in experiment CP5 with FR (Table 4): after 2 months, 10.1 mM of acetate, corresponding to a potential production of 20.2 mM of bicarbonates, had been transformed whereas only 6.4 mM of bicarbonates had been produced (Table 4). The bicarbonate increase is therefore not sufficient to explain the acetate decrease (Table 4). One explanation is that acetate can be used by dissimilatory bacteria not only as an energy source (oxidation) but also as a carbon source (biosynthesis).

Other explanations for the delayed solubilization of arsenic were also omitted in the modeling: thus it might be possible that the microbiological As reducing factor is expressed only in the presence of As(V) in solution or, conversely, that the reduction of As(V) occurs on the surface of 2LFh after sorption of Fe(II). In the first case, a prerequisite for As(V) reduction is the release of As(V) without re-adsorption; in the second case, it is a reduction of solid Fe(III) or the release and re-adsorption of Fe(II). The abiotic case, i.e. the reduction of As(V) coupled to the oxidation of adsorbed Fe(II), has indeed been observed with the reduction of U(VI) by surface Fe(II) [49,50,60]. Moreover, a recent article concerning the re-oxidation of uranium effectively showed that both As(V) reduction theories (biotic and abiotic) are not mutually exclusive as mechanisms of As mobilization [61].

It may also be possible that released As above a certain threshold is toxic for DIRB, which could explain why iron-reducing bacteria became metabolically inactive after one month and only arsenic-reducing organisms maintained their metabolic activity.

## Conclusion

The experimental and modeling results with synthetic As-2LFh in the presence of a community of metal-reducing bacteria have significant implications with regard to the mobility of arsenic in groundwater, particularly in the Bengal Delta Plain. Recent incubations of sediments from Bengal have clearly shown that arsenic is "strongly adsorbed on or incorporated in the predominantly Fe(III) oxyhydroxides" [11]. One hypothesis is that the transport and the delivery of organic carbon from surface to subsurface bacterial communities may play a key role in enhancing arsenic mobility by reducing a significant proportion of Fe(III) hydroxides [3,6]. We provide further convincing evidence with our experiments that direct biotic reduction of As(V) may also explain why a limited release of iron (e.g. 3%) can be followed by a more significant release of arsenic (e.g. 9%), as was recently observed with the incubation of Bengali sediments [11,12].

The biogeochemical model developed to simulate these results in both the aqueous and solid phases provides us with two main conclusions: (1) 2LFh reduction is energetically more favorable than arsenic reduction at the beginning of the incubations, and (2) once arsenic reduction is energetically possible, the release of As is due to its reduction to more weakly adsorbed As(III). A sensitivity analysis of this model has shown that the mobility of arsenic may be controlled by the competition of arsenite with carbonate species for sorption onto ferrihydrite, i.e. the two by-products of dissimilatory As(V) reduction.

The model was also successfully applied to recent experimental results on the release of arsenic from Bengal delta sediments [12]. We therefore suggest that competition between the two by-products of dissimilatory As(V) reduction, arsenite As(III) and carbonate species, may also control the mobilization of arsenic in alluvial aquifers, such as those of the Bengal delta. Additional research is nevertheless required to determine the role of other competitors such as silicates and phosphates and of other sorbents such as iron sulfides. The biogeochemical model approach may, in the future, be a significant aid for understanding the mobility of arsenic associated with iron oxides in subsurface environments and in predicting its behavior under different management scenarios.

## Supplementary Material

Additional file 1 Composition of growth media and phylogenetic characterization. The data provided describe the both growth media and the phylogenetic affiliation of the pure strains which were isolated from the FR bacterial community. Click here for file
