# Supplementary material for: Decoupling of arsenic and iron release from ferrihydrite suspension under reducing conditions: a biogeochemical model
Source: Geochem Trans. 2007 Nov 29;8:12. doi: 10.1186/1467-4866-8-12 (PMC2246110; doi:10.1186/1467-4866-8-12)
Supplement: Additional file 1 — Composition of growth media and phylogenetic characterization. The data provided describe the both growth media and the phylogenetic affiliation of the pure strains which were isolated from the FR bacterial community. [file 1467-4866-8-12-S1.doc]

**Additional file 1: Composition of growth media and phylogenetic characterization**

## Selection of iron-reducing bacteria (FR) with the Bromfield medium

The bacterial community FR was selected from a soil containing high concentrations of heavy metals without contamination with As (industrial site). FR was selected for its ability to reduce Fe(III) to Fe(II) in controlled conditions at pH 6. A modified Bromfield medium (BR) was used for selection and maintenance. The composition of BR growth medium is available in Table S1.

Table S1

| **Bromfield medium composition (ref. [1])** |  |
| --- | --- |
| Component | Quantity |
| Glucose | 9 g |
| Yeast Extract | 0.15 g |
| KH_2_PO_4_ | 0.5 g |
| (NH_4_)_2_SO_4_ | 1 g |
| MgSO_4_, 7H_2_O | 0.5 g |
| CaCO_3_ | 4 g |
| Amorphous oxyhydroxide of iron (HFO) | 0.5 g |
| Deionized water | 1000 mL |

Particles of HFO from BR, which were prepared as described in Materials and Methods but without addition of arsenic were poured together into a hermetically closed glass flask and autoclaved three times for 1 hour at 110°C at 24 hours intervals. Selection of the FR community was achieved by incubating 70 g of soil in 500 mL of BR medium containing HFO under a CO_2_ atmosphere in a 1 L bottle. After one month of incubation, 10 mL of this enrichment were transferred to a 250 mL CO_2_-tight flask containing 90 mL of BR medium.

The BR medium which contained glucose was only used for initial selection and maintenance of a microbial population, including probably dissimilatory iron-reducing bacteria (DIRB) and/or fermenting bacteria. Because glucose is a well known fermentable carbon source, we used for all experiments a different culture medium called CAsR1 with acetate and lactate instead of glucose as carbon sources, in order to select mainly DIRB rather than fermenting bacteria.

## Composition of growth medium CAsR1 used in experiments

A growth medium was used previously for growing arsenic-reducing bacteria by Battaglia et al. (2002) [2]. It contains mainly acetate and lactate and also trace elements (Table S2). It was used for all experiments AD1, CP1, AD5, CP5.

Table S2

| **CAsR1 medium composition (ref. [2])** |  |
| --- | --- |
| Component | Quantity |
| Na-Lactate | 2.2 g |
| Na-Acetate, 3H_2_O | 2.0 g |
| KH_2_PO_4_ | 0.2 g |
| NaCl | 0.5 g |
| NH_4_Cl | 0.2 g |
| MgCl_2_, 6H_2_O | 0.1 g |
| CaCl_2_, 2H_2_O | 0.1 g |
| *Trace elements solution^*^* | 1 mL |
| Deionized water | 1000 mL |

**Trace elements solution*

| HCl solution (25%) | 6.5 mL |
| --- | --- |
| FeCl_2_, 4H_2_O | 1.5 g |
| H_3_BO_3_ | 60 mg |
| MnSO_4_, H_2_O | 117 mg |
| CoCl_2_, 6H_2_O | 25 mg |
| ZnCl_2_ | 70 mg |
| NiCl_2_, 6 H_2_O | 25 mg |
| CuCl_2_, 2H_2_O | 15 mg |
| Na_2_MoO_4_, 2 H_2_O | 25 mg |
| Deionized water | 1 000 mL |

## Experimental method for isolation of pure strains from FR with phylogenetic characterization

Five successive dilutions of FR community were performed in sterile deionized water and 0.1 mL of each dilution were inoculated on Petri dishes containing solid Bromfield medium. Incubations were performed at 25°C in anaerobic boxes containing an oxygen trap (Anaerocult Merck). Different types of isolated colonies were inoculated separately in 10 mL of Bromfield medium and immediately transferred to a 250 mL CO_2_-tight flask containing 90 mL of Bromfield medium and 0.05 g of HFO. To conserve each pure strain, 10 mL of the cultures were monthly transferred into the same medium.

Genomic DNA was extracted from cell pellets by bead beating using the FastDNA® MH Kit and the Bio101 FastPrep® Instrument (Bio101, Vista, Ca.). Small-subunit ribosomal RNA (16S rRNA) genes were amplified by PCR using the bacterial primers 9F (5’-AGAGTTTGATCCTGGCTCAG-3’) and 1492R (5’-TACGGTTACCTTGTTACGAC-3’), an annealing temperature of 55°C, and 30 cycles. PCR products were purified with the GeneClean Turbo Kit (Qbiogene, Montreal, Canada) and cloned with the pGEM-T Easy Cloning Kit (Promega, San Luis Obispo, Calif.). Plasmids containing the insert of the right length (ca. 1.5 kb) were purified using the Wizard Plus SV Miniprep DNA Purification Kit (Promega). Inserts were sequenced by Genome Express (Grenoble, France) with primers SP6 (5’-ATTTAGGTGACACTATAGAA-3’) and T7 (5’-TAATACGACTCACTATAGGG-3’) targeting the plasmid. Partial 16S rRNA sequences were aligned with reference sequences using the sequence editor BioEdit [3]. Only unambiguously aligned sequence positions (1415 nucleotides) were exported to Treecon version 1.3b program [4], and distance analyses were performed by the method of Jukes and Cantor [5]. 16S rRNA gene sequences have been deposited to GenBank under accession numbers AY925091, AY925092 and AY925093.

The capacity of the pure strains as well as the FR community to use As(V) as terminal electron acceptor was tested in the selective medium for dissimilatory As(V)-reducing bacteria described by Kuai et al. [6].

## Phylogeny of pure strains isolated from FR

After one week of incubation at 25°C in anaerobic boxes, three morphologically different types of colonies (FRB1, FRB2 and J) were observed and maintained in axenic cultures. Among these pure strains, only FRB1 was able to reduce aqueous As(V) to As(III) under anaerobic conditions. Phylogenetic analyses of 16S rRNA gene sequences (1415 bp) clearly affiliated strains FRB1 and J to the Gram-positive genus *Clostridium*, and to the species *C. pasteurianum* and *C. tyrobutyricum*, respectively (> 99% identities). Strain FRB2 belonged to the Gram-positive genus *Sporolactobacillus*, being most closely related to *S. nakayamae* subsp. *nakayamae* (> 99.5% identities).

Based solely on 16S rRNA phylogeny, none of the isolated strain can be considered as a new species, identity values being higher than the recommended limit of 97% for species definition [7].

## Phylogenetic affiliation of the isolated strain FRB1

See Figure S1

Figure S1 : Phylogenetic affiliation of FRB1 isolated from FR community (which respires Fe(III) and As(V)). The 16S rDNA sequence of *Clostridium proteolyticum* was used to root the tree.

There are recent reports on the implication of Clostridium species in As(V)-reducing activity: sp. OhilAs [8], ARCL1 and AKAR3 [9]. However, the 16S RNA sequences of these species were not available in databases and could not be compared to the sequence of strain FRB1.

**References**

1. Bousserrhine N. *PhD thesis.* Univ. Nancy 1995.

2. Battaglia-Brunet F, Dictor M, Garrido F, Crouzet C, Morin D, Dekeyser K, Clarens M, Baranger P: *Journal of applied microbiology* 2002, **93:**656-667.

3. Hall TA: *Nucleic acids Symp* 1999, **41:**95-98.

4. Van de Peer Y. and De Wachter R: *Comp Applic Biosci* 1994, **10:**569-570.

5. Jukes TH and Cantor CR: **Evolution of protein molecules.** In *Mammalian Protein Metabolism* Edited by Munro H. N., New York: Academic Press; 1969:211-232.

6. Kuai L, Nair AA, Polz MF: *Appl.Environ.Microbiol.* 2001, **67:**3168-3173.

7. Wayne LG, Brenner DJ, Colwell RR, Grimont PAD, Kandler O, Krichevsky MI, Moore LH, Moore WEC, Murray RGE: *Int J Syst Bacteriol* 1987, **37:**463-464.

8. Oremland RS and Stolz JF: The ecology of arsenic. Science 2003, 300:939-944.

9. Rhine ED, Garcia-Dominguez E, Phelps CD, Young LY: Environmental Microbes Can Speciate and Cycle Arsenic. Environmental Science & Technology 2005, 39:9569-9573.
